# Global Reorganization of Replication Domains During Embryonic Stem Cell Differentiation

**DOI:** 10.1371/journal.pbio.0060245

**Published:** 2008-10-07

**Authors:** Ichiro Hiratani, Tyrone Ryba, Mari Itoh, Tomoki Yokochi, Michaela Schwaiger, Chia-Wei Chang, Yung Lyou, Tim M Townes, Dirk Schübeler, David M Gilbert

**Affiliations:** 1 Department of Biological Science, Florida State University, Tallahassee, Florida, United States of America; 2 Friedrich Miescher Institute for Biomedical Research, Basel, Switzerland; 3 Department of Biochemistry and Molecular Genetics, University of Alabama at Birmingham, Schools of Medicine and Dentistry, Birmingham, Alabama, United States of America; 4 Department of Biochemistry and Molecular Biology, State University of New York, Upstate Medical University, Syracuse, New York, United States of America; Friedrich Miescher Institute for Biomedical Research, Switzerland

## Abstract

DNA replication in mammals is regulated via the coordinate firing of clusters of replicons that duplicate megabase-sized chromosome segments at specific times during S-phase. Cytogenetic studies show that these “replicon clusters” coalesce as subchromosomal units that persist through multiple cell generations, but the molecular boundaries of such units have remained elusive. Moreover, the extent to which changes in replication timing occur during differentiation and their relationship to transcription changes has not been rigorously investigated. We have constructed high-resolution replication-timing profiles in mouse embryonic stem cells (mESCs) before and after differentiation to neural precursor cells. We demonstrate that chromosomes can be segmented into multimegabase domains of coordinate replication, which we call “replication domains,” separated by transition regions whose replication kinetics are consistent with large originless segments. The molecular boundaries of replication domains are remarkably well conserved between distantly related ESC lines and induced pluripotent stem cells. Unexpectedly, ESC differentiation was accompanied by the consolidation of smaller differentially replicating domains into larger coordinately replicated units whose replication time was more aligned to isochore GC content and the density of LINE-1 transposable elements, but not gene density. Replication-timing changes were coordinated with transcription changes for weak promoters more than strong promoters, and were accompanied by rearrangements in subnuclear position. We conclude that replication profiles are cell-type specific, and changes in these profiles reveal chromosome segments that undergo large changes in organization during differentiation. Moreover, smaller replication domains and a higher density of timing transition regions that interrupt isochore replication timing define a novel characteristic of the pluripotent state.

## Author Summary

Microscopy studies have suggested that chromosomal DNA is composed of multiple, megabase-sized segments, each replicated at different times during S-phase of the cell cycle. However, a molecular definition of these coordinately replicated sequences and the stability of the boundaries between them has not been established. We constructed genome-wide replication-timing maps in mouse embryonic stem cells, identifying multimegabase coordinately replicated chromosome segments—“replication domains”—separated by remarkably distinct temporal boundaries. These domain boundaries were shared between several unrelated embryonic stem cell lines, including somatic cells reprogrammed to pluripotency (so-called induced pluripotent stem cells). However, upon differentiation to neural precursor cells, domains encompassing approximately 20% of the genome changed their replication timing, temporally consolidating into fewer, larger replication domains that were conserved between different neural precursor cell lines. Domains that changed replication timing showed a unique sequence composition, a strongly biased directionality for changes in resident gene expression, and altered radial positioning within the three-dimensional space in the cell nucleus, suggesting that changes in replication timing are related to the reorganization of higher-order chromosome structure and function during differentiation. Moreover, the property of smaller discordantly replicating domains may define a novel characteristic of pluripotency.

## Abstract 3

Analyzing the temporal order of DNA replication across the genome during embryonic stem cell differentiation reveals stable boundaries between coordinately replicated regions that consolidate into fewer, larger domains during differentiation.

## Introduction

Despite our rapidly expanding knowledge of the structure and function of eukaryotic chromatin at the individual nucleosome level, little is known about the higher-order organization of chromosomes [1]. DNA replication provides an excellent forum with which to investigate these levels of chromosome organization. Large segments of chromosomes replicate coordinately, mediated by the nearly synchronous firing of clusters of replication origins (“replicon clusters”) at specific times during S-phase ([2] and references therein). Replicon clusters can be visualized in living cells as discrete foci by pulse labeling with fluorescent nucleotide analogs. When followed through multiple cell divisions, labeled foci do not mix, separate, or change in shape, indicating that the DNA that replicates coordinately derives from a single chromosome segment [3–6]. Moreover, replicon clusters that fire at different times during S-phase occupy different subnuclear compartments, with early-replicating foci showing enrichment in the nuclear interior, whereas foci replicating later during S-phase are enriched in perinucleolar regions and the nuclear periphery [4,7]. The order in which these segments replicate is established during early G1-phase, coincident with the establishment of their subnuclear positions after nuclear reassembly [4,8]. Together, these results support the hypothesis that coordinately replicated segments of chromosomes form stable units of chromosome structure and nuclear architecture that are maintained from cell cycle to cell cycle. However, the data supporting this model are mainly cytogenetic; molecular evidence for stable replication-timing boundary sequences has been difficult to obtain.

Several studies have found that regions of coordinate replication timing correspond to regions of alternating GC content, or isochores, with GC-rich regions replicating early and AT-rich regions replicating late [9–11], leading some to conclude that replication timing is a relatively static chromosomal feature conserved in all cell types [12,13]. However, replication timing cannot be dictated by sequence alone, as both genomic imprinting and X chromosome inactivation are accompanied by the asynchronous replication of homologs [14,15]. Moreover, the replication-timing program for at least some regions is different in different tissues [16–21], and changes in replication timing can be detected during the course of differentiation [22,23]. These differences appear to be related to differential gene expression since there is a strong positive correlation between early replication and transcriptional activity in cultured *Drosophila* [24,25] and human cell lines [9,10,26], and genes generally replicate earlier when transcriptionally active. This relationship is not direct; instead, early replication appears to be associated with a chromatin state that is permissive for transcription [16]. Nonetheless, it is not clear how much of the genome ever changes replication timing. A comparison of human Chromosome 22 between fibroblast and lymphoblastoid cells revealed that only 1% of this chromosome differed in replication time [9], whereas analyses of individual genes revealed that changes during differentiation were restricted to a subset of genes residing within AT-rich isochores [22]. Hence, determining the extent to which replication timing changes occur during differentiation is a fundamental unresolved question.

Major cell fate changes occur early in development when pluripotent cells commit to specific germ layers. Loss of pluripotency can be recapitulated through the differentiation of embryonic stem cells (ESCs), which has been associated with changes in the dynamics of chromatin [27], posttranslational modifications of histones, and nuclear architecture [28]. By focusing on this specific developmental period, we wished to analyze the extent of replication-timing changes genome-wide to address whether replication timing is a static or dynamic property of chromosomes during the course of differentiation. Here, we performed genome-wide analyses of three mouse ESC (mESC) lines before and after differentiation to neural precursor cells (NPCs). Replication domain organization was highly conserved between all three ESC lines and with fibroblasts that were induced to the pluripotent state (induced pluripotent stem cells [iPS cells]). However, 20% of the genome showed substantial changes in replication timing upon neural differentiation. Intriguingly, differentially replicating domains in ESCs consolidated to generate larger coordinately regulated units in NPCs that showed a considerably higher degree of alignment of replication timing to isochore sequence composition. We conclude that replication domain organization is highly dynamic, including its relationship to isochores, and we provide evidence that smaller replication domains that disrupt the alignment of replication timing to isochores define a novel characteristic of the pluripotent state. Furthermore, our findings suggest that DNA replication may provide a molecular handle on the study of previously impenetrable levels of higher-order chromosomal organization.

## Results

### Replication Domain Structure in Embryonic Stem Cells

**Figure 1:**
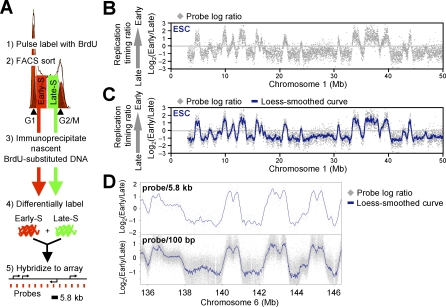
Genome-Wide Analysis of Replication Timing in mESCs (A) Protocol for genome-wide replication timing analysis using oligonucleotide microarrays with one probe every 5.8 kb. (B and C) Generating replication-timing profiles. An exemplary mESC replication-timing profile of a Chromosome 1 segment is shown. Raw values for probe log ratios [i.e., log_2_(Early/Late)] along the chromosome revealed a clear demarcation between regions of coordinate replication (B), which is highlighted upon overlaying a local polynomial smoothing (loess) curve (C). (D) Analyses at a density of one probe per 5.8 kb or 100 bp show essentially identical smoothed replication-timing profiles.

The genome-wide analysis of replication timing in mammalian cells has been reported for only one cell type at a density of one probe per megabase [10], which was not sufficient to evaluate the extent to which the genome is organized into coordinately replicating domains. Hence, we mapped replication timing in mESCs using high-density oligonucleotide arrays, adapting a previously developed retroactive synchronization method [24,29]. ESCs were chosen because they provide the opportunity to directly evaluate dynamic changes in the replication program in response to changes in growth conditions [22,23], in contrast to comparisons of separately isolated cell lines that may harbor genetic differences or long-term epigenetic adaptations. Cells were pulse labeled with BrdU and separated into early and late S-phase fractions by flow cytometry (Figure 1A). BrdU-substituted DNA from each fraction was immunoprecipitated with an anti-BrdU antibody, differentially labeled, and cohybridized to a mouse whole-genome oligonucleotide microarray (Nimblegen Systems) (Figure 1A). The ratio of the abundance of each probe in the early and late fraction [“replication timing ratio” = log_2_(Early/Late)] was then used to generate a replication-timing profile for the entire genome at a density of one probe every 5.8 kb. Replicate experiments in which early- and late-replicating DNA were reciprocally labeled (“dye-switch”) showed a high degree of correlation and were averaged (*R* ^2^ values ranged between 0.86 and 0.95 after loess smoothing). Datasets were confirmed by PCR analysis of 18 genes (100% consistent) and by comparison to two previously published replication-timing analyses of 90 individual genes in mESCs (91% consistent) [22,23] (Figure S1A–S1C).

Figure 1B shows the mean replication-timing ratio for each probe plotted as a function of chromosomal coordinate for an exemplary 50-Mb segment of Chromosome 1, and Figure 1C shows a loess-smoothed curve fit for the same region. This profile revealed a surprisingly clear demarcation between regions of coordinate replication that we heretofore refer to as “replication domains.” To address whether 5.8-kb probe density was sufficient to provide a complete profile of replication domains, we hybridized the same duplicate preparations of replication intermediates to tiling microarrays (one probe every 100 bp) of Chromosomes 6 and 7. Despite the nearly 60-fold–higher probe density, results showed an almost indistinguishable smoothed profile (Figure 1D). This is consistent with known properties of DNA replication; a 2-h BrdU pulse is expected to label 200–400-kb stretches of DNA (fork rate 1–2 kb/min; [6,30,31]), and since multiple replicons across hundreds of kilobases fire synchronously (reviewed in [2]), probes spaced 5.8 kb apart would be expected to replicate at very similar times. Indeed, high autocorrelation of replication timing between neighboring probes extends over 1 Mb (Figure S2). Hence, replication timing across the entire genome can be reliably profiled on a single oligonucleotide chip. Replication profiles for all chromosomes are displayed on our Web site and are available for downloading: http://www.replicationdomain.org.

**Figure 2:**
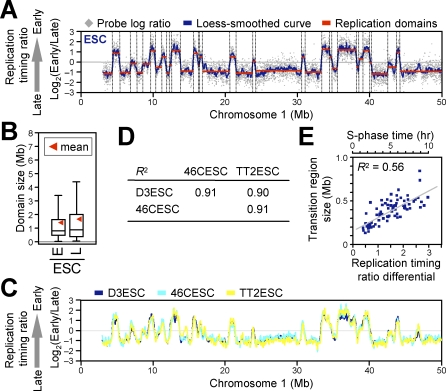
Replication Domain Structure and Its Conservation between Three Independent mESC Lines (A) Identification of replication domains (red lines) and their boundaries (dotted lines) by a segmentation algorithm [32]. (B) Box plots of early (E; log ratio > 0) and late (L; log ratio < 0) replication domain sizes. Horizontal bars represent the 10th, 25th, 50th (median), 75th, and 90th percentiles, respectively. (C and D) Three different mESC lines (D3, 46C, and TT2) showed remarkably similar replication domain organization, as revealed by visual inspection of a segment on Chromosome 1 (C) and high Pearson *R* ^2^ values for pair-wise comparisons (D). (E) A scatter plot of replication-timing differential versus physical distance (Mb) between the ends of 75 randomly chosen pairs of adjacent replication domains (replication-timing transition regions) from Chromosomes 2, 11, and 16 (25 each) revealed a positive correlation with a slope that is consistent with mammalian replication fork speeds. A time scale is provided, based on the assumption that replication-timing ratio difference of three roughly corresponds to an approximately 10-h S-phase.

To quantify the numbers and positions of replication domains and their boundaries genome-wide, we adapted a segmentation algorithm—originally developed to identify copy number differences for comparative genomic hybridization [32]—to identify regions of uniform *y*-axis values (see Materials and Methods), which are illustrated in Figure 2A. This algorithm generates a dataset consisting of the nucleotide map positions for the boundaries of each replication domain. Domain sizes ranged from 200 kb to 2 Mb, with some considerably larger domains (Figures 2B and S3A). These domain sizes provide a logical explanation as to why existing ENCODE replication-timing data for HeLa cells [33] does not reveal replication domains; the ENCODE regions cover 1% of the genome and consist primarily of scattered 500-kb genomic segments, which would be too small to discern replication domain–level chromosome organization. It is also possible that the genetic and epigenetic instability of HeLa cells contributed to the blurring of domain boundaries. Domains were found to replicate at all times during S-phase, however, domains larger than 2.5 Mb were either very early or very late replicating, suggesting that coordinately replicating regions larger than a certain threshold size tend to replicate at one extreme or another of S-phase (Figure S3D). Our results were not an artifact of probe density, segmentation algorithm, or synchronization method; similar distributions were obtained with a density of one probe per 100 bp, using different segmentation parameters, and using an alternative protocol [10] that determines replication timing by probe copy number in S-phase versus G1-phase, without fractionation of S-phase (unpublished data). Similar results were also obtained with human ESCs (hESCs; J. Lu, I. Hiratani, T. Ryba, and D. M. Gilbert, unpublished data).

### Domain Structure Is Conserved between Independent mESC Lines

The results described above demonstrate that coordinately replicated regions (replication domains) constitute functional units of chromosomes whose boundaries can be molecularly defined. The fact that we can so precisely map replication domain boundaries in populations of cells demonstrates that their positions are highly stable from cell cycle to cell cycle. To evaluate whether these boundaries are a conserved property of chromosomes in multiple mESCs, we compared three mESC lines from two independently established mouse inbred strains. Lines D3 and 46C were both derived from the 129 mouse strain and so are nearly identical genetically but separated by more than 20 y in cell culture, whereas TT2 was derived 15 y ago from a C57BL/6xCBA hybrid mouse and is therefore genetically polymorphic [34–36]. Despite the disparate genetic and temporal histories of these three cell lines, their replication profiles were virtually identical (Figure 2C and 2D). This demonstrates that replication domain structure is a highly conserved property of mESCs. Moreover, the recent demonstration that mESCs display considerable cell-to-cell heterogeneity in the expression of certain pluripotency-specific marker genes such as *Nanog* and *Rex1* [37,38] indicates that replication-timing profiles are a substantially more stable and homogeneous property of ESCs than transcription profiles.

### Transitions between Replication Domains Are Consistent with Large Originless Regions of Unidirectional Replication

Our results demonstrate that replication timing is regulated at the level of large domains that replicate coordinately, separated by noticeable transition regions. These transition regions resemble the originless transition between early- and late-replicating segments of the immunoglobulin IgH locus [30], where a unidirectional replication fork travels 450 kb. If such transition regions throughout the genome represent unidirectional forks, which in mammalian cells travel at the rate of 1–2 kb per minute [6,30,31], then we would expect a linear relationship between the time and distance between each replication domain. We examined the transitions between 25 randomly selected replication domain boundaries each from Chromosomes 2, 11, and 16 (total of 75; see Materials and Methods). For each of these boundaries, we scored both the replication-timing ratio difference and the kilobase distance from the distal “ledge” of one domain to the proximal “ledge” of the next (see Materials and Methods), and plotted these relative to each other (Figure 2E). Indeed, there was a clear positive linear correlation between the distance and time between replication domains, with boundaries ranging in size from 0.1 to 0.6 Mb. Since the replication-timing ratios for the entire dataset ranged from approximately −1.5 to +1.5, which represents an approximately 10-h S-phase, we estimate that a unidirectional fork would need to travel 1.4 kb/min on average (ranging from 0.8 to 3.5 kb/min), which is consistent with mammalian replication fork speeds. Given this linear relationship and the uniform slope of each transition region, our data strongly suggest that the boundaries between replication domains define originless regions of unidirectional replication throughout the genome. Regions where individual replication forks need to travel long distances may delineate genomic regions that are particularly vulnerable to DNA damage since stalled forks can form reactive recombination intermediates that lead to chromosome rearrangements [39]. In fact, a survey of a few such boundaries correlated them with genes that are frequently disrupted in cancer [40,41].

### Replication Domain Profiles Change in A Characteristic Way during Neural Differentiation

**Figure 3:**
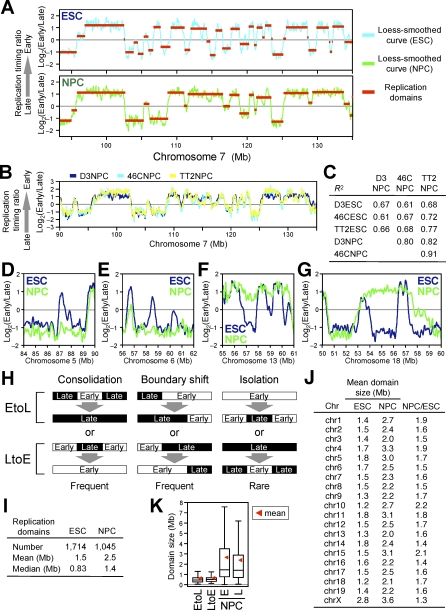
Global Reorganization of Replication Domains upon Differentiation of ESCs to NPCs (A) Replication domain profiles change dramatically upon differentiation of ESCs to NPCs. An exemplary Chromosome 7 segment is shown. (B and C) NPCs derived from distinct neural differentiation schemes and three independent mESC lines showed fairly similar replication timing profiles, both by visual inspection (B) and high Pearson *R* ^2^ values for pair-wise comparisons of NPCs (C). The low *R* ^2^ values for pair-wise comparisons of ESCs and NPCs confirm that substantial changes occurred upon differentiation (C). (D–G) Exemplary EtoL (Early-to-Late) (D and E) and LtoE (Late-to-Early) (F and G) consolidation. (H) Schematic representation of domain consolidation, boundary shift, and isolation. (I) Summary of replication domain properties in ESCs and NPCs. (J) Replication domain sizes by chromosome. Chromosome Y was underrepresented on the microarray and was excluded from the analysis. (K) Box plots of sizes of domains that changed replication timing (EtoL and LtoE), as well as early- and late-replicating domains in NPCs. EtoL and LtoE domains show smaller and tighter distribution than domains in NPCs or ESCs (Figure 2B).

If replication timing is regulated during development but is stable within a particular cell type, then replication domain maps could represent cell-type–specific “epigenetic signatures.” As discussed in the introduction, the extent to which replication timing may differ in different cell types is currently not clear, and some studies have concluded that there are few if any differences between cell types [9,12,13]. To directly address the extent to which replication-timing changes occur during the course of differentiation, we generated replication profiles following differentiation of ESCs to NPCs using two different neural differentiation protocols: one that used a conditioned medium to differentiate D3 ESCs as embryoid bodies [42], and one that used a chemically defined medium to differentiate 46C and TT2 ESCs in adherent monolayers [36]. Results revealed substantial changes in the replication profile (Figure 3A): even after excluding regional differences of fewer than nine consecutive probes (52 kb), 20% of probes showed a log ratio change of more than 0.5, as compared to 3% of probes showing differences either between ESC lines or between neural differentiation protocols. Importantly, replication profiles for NPCs were similar regardless of the ESC line or neural differentiation protocol employed (Figure 3B and 3C) and despite differences in the levels of certain gene expression markers between the differentiated cell populations produced by these two protocols (unpublished data). This demonstrates that the observed changes are characteristic of NPCs rather than having been elicited by conditions associated with a particular neural differentiation protocol (albeit there are more differences between NPCs than between ESCs). We conclude that specific changes in replication timing take place during the course of neural differentiation, generating a novel replication profile that is characteristic of NPCs, suggesting that replication-timing profiles are stable within particular cell lineages but change significantly in response to major cell fate decisions.

### Global Replication Domain Consolidation during Differentiation

Unexpectedly, we found that replication-timing changes induced by differentiation resulted in a dramatic change in the number and sizes of replication domains (Figure 3A). Small domains that were replicated at different times in ESCs frequently merged to become one larger coordinately replicated domain (Figure 3D–3G). We refer to this reorganization as domain “consolidation” (Figure 3H). Also frequent were events in which the positions of boundaries shifted (referred to as a “boundary shift”). Boundary shifts occurred equally through the encroachment of late domains into early domains and vice versa, so did not affect the overall size or number of replication domains. In rare cases, we observed the emergence of new smaller domains from within a larger domain (referred to as “isolation”) (Figure 3H). Visual inspection of 46 domains that changed replication timing (22 LtoE [Late in ESCs to Early in NPCs] and 24 EtoL [Early-to-Late]) confirmed that “consolidation” and “boundary shift” events were equally frequent (43% and 50%, respectively), whereas “isolation” events were rare (7%). Domain consolidation was significant, with a 40% reduction in the number of domains and a corresponding increase in the size of domains (Figures 3I, S3A, and S3B). Importantly, consolidation was widespread, occurring on all chromosomes (Figure 3J). Interestingly, domains that switched replication timing (EtoL and LtoE) were smaller and more uniform in size (400–800 kb) than the distribution of domains as a whole (Figures 3K and S3C). This size range is very close to cytogenetic estimates of the amount of DNA within individual replication foci [5]. This suggests that replication domains are made up of smaller units that may correspond to replication foci or “replicon clusters” and that replication timing changes may occur at the level of these smaller units (see Discussion). Together, these results demonstrate a global reorganization and consolidation of replication domains during ESC differentiation.

### Consolidation Aligns Replication Domains to Isochore GC Content

**Figure 4:**
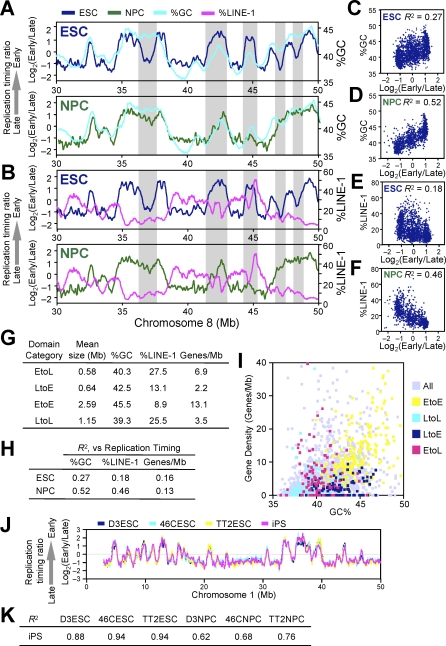
Domain Consolidation Aligns Replication Timing to GC/LINE-1 Content but Not Gene Density (A and B) Loess-smoothed replication-timing profiles of ESCs (blue) and NPCs (green) were overlaid with moving averages of 500-kb windows of GC (A) and LINE-1 (B) content for a segment on Chromosome 8. Grey highlighted areas show regions where differentiation aligns replication timing to GC/LINE-1 content. (C–F) Average replication-timing ratios of replication domains in ESCs (C and E) and NPCs (D and F) were plotted against their GC content (C and D) and LINE-1 content (E and F). Pearson's *R* ^2^ values are shown. (G) EtoL and LtoE domains have an unusual combination of GC/LINE-1 content and gene density. Domains with the 5% greatest replication-timing changes were defined as EtoL and LtoE, whereas those with the least changes (lowest 20%) that maintained replication timing ratio of above 0.5 or below −0.5 were defined as EtoE and LtoL, respectively. Genes/Mb refers to RefSeq gene density. (H) Correlation of gene density and replication timing at the level of domains in ESCs and NPCs. Pearson *R* ^2^ values are shown. (I) Scatter plot of GC content and gene density shows that EtoE, LtoL, LtoE, and EtoL domains are generally GC rich/gene rich, GC poor/gene poor, GC rich/gene poor, and GC poor/gene rich, respectively. (J and K) Replication domain structure of iPS cells matches that of ESCs both by visual inspection (J) and high Pearson *R* ^2^ values for pair-wise comparisons of iPS to ESCs (K).

Mammalian chromosomes are organized into alternating AT- and GC-rich stretches of sequence called isochores, which are rich and poor in LINE-1 transposable elements, respectively [43]. Prior studies evaluating replication timing of various segments of the human genome reported a strong positive correlation between GC content and early replication [10,11,13,41]. Our analysis also detected such a correlation (Figure 4), but the degree of this correlation was not static. In fact, the correlation between replication domains and isochores was not impressively strong in mESCs but improved substantially during differentiation. This was evident by visual comparison of replication profiles to GC and LINE-1 density in ESCs versus NPCs (Figure 4A and 4B). To confirm this alignment genome-wide, the GC or LINE-1 content of the DNA sequences within the boundaries of each replication domain was plotted versus the replication time of each domain. For both sequence properties, the correlation became much stronger in NPCs versus ESCs (Figure 4C–4F). Moreover, domains that changed replication timing usually acquired a temporal profile in line with their isochore sequence composition: in other words, EtoL domains were low in GC and high in LINE-1 density and resembled LtoL (Late-to-Late) domains, while LtoE domains had an intermediate GC content and a relatively low LINE-1 density and resembled EtoE (Early-to-Early) domains (Figure 4G).

### Domains that Change Replication Timing Have an Unusual Sequence Composition

GC- versus AT-rich isochores are also known to be gene rich versus gene poor [44]. As expected, gene density within replication domains largely followed the rules of isochore replication timing: in both ESCs and NPCs, domains that had a high density of genes were early replicating and, for the most part, GC rich. In fact, 75% of genes were replicated early in both cell types (i.e., positive replication-timing ratios) and, as expected, EtoE and LtoL domains were GC rich/gene rich and GC poor/gene poor, respectively (Figure 4G). Surprisingly, although the alignment to isochore GC/LINE-1 density increased during differentiation, the correlation between gene density and early replication did not (Figure 4H). This was due to the fact that LtoE and EtoL domains exhibited the unusual properties of being GC rich/gene poor and GC poor/gene rich, respectively (Figure 4G and 4I). Thus, GC/LINE-1 density and gene density are properties of isochores that can be uncoupled. Moreover, these results demonstrate that replication timing is not a simple reflection of either local gene density or isochore GC content, as has been proposed by other studies that relied on smaller datasets [12,13]. We conclude that segments that change replication timing have an unusual combination of GC content and gene density, providing a potential means to predict chromosome domains that change replication timing.

### Replication Domain Structure of Induced Pluripotent Stem Cells Matches that of ESCs

Our results described above suggest that replication-timing profiles in ESCs could provide a unique signature or “fingerprint” for the pluripotent state. A prediction of this hypothesis is that iPS cells, in which an adult differentiated cell has been reverted back to the pluripotent state, should share replication profiles with ESCs. To address this prediction, we generated replication profiles for iPS cells [45], which were reprogrammed from tail-tip fibroblasts derived from a 129xBL-6 hybrid strain of mice as described [46]. Indeed, iPS cells showed a profile that was virtually indistinguishable from other ESCs (Figure 4J and 4K). These results provide additional evidence that iPS cells are indeed very similar to ESCs and that the property of smaller replication domains that disrupt the alignment of replication timing to isochores is a novel characteristic of the pluripotent state. Moreover, our results suggest a means to profile or “fingerprint” cell types, including pluripotent cell types, based on replication domain organization, which appears to be considerably more stable than transcription profiles.

### Replication Timing and Transcription Changes during Differentiation

#### Correlation between early replication and transcription is similar in ESCs and NPCs.

**Figure 5:**
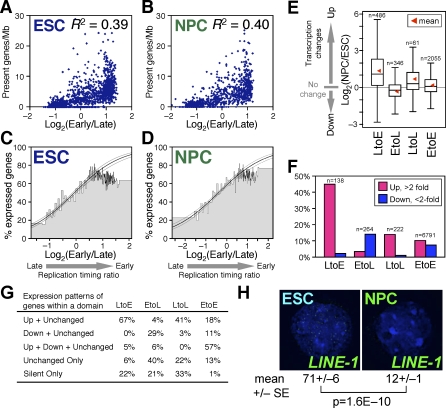
Replication Timing and Transcription Changes during Differentiation (A and B) Average replication-timing ratios of replication domains were plotted against their “present” (i.e., transcriptionally active) gene density for ESCs (A) and NPCs (B). Pearson *R* ^2^ values are shown. (C and D) Correlation between early replication and the probability of expression. Genes were ranked by their replication-timing ratio and divided into bins of 100 genes, the height of which represents the percentage of active (i.e., ”present”) genes within each bin. The width and position of each bin represents its range of replication-timing ratios. Logistic regression (inner line) and 95% confidence intervals (outer lines) reveal a clear correlation in both ESCs (C) and NPCs (D). By the Likelihood Ratio test (a goodness-of-fit test), the fitted model is significantly different (*p* < 2 × 10^−16^ for both ESCs and NPCs) from that of a null hypothesis in which replication timing has no correlation to transcription. (E) Box plots showing the fold changes in transcription [i.e., log_2_(NPC/ESC)] of LtoE, EtoL, LtoL, and EtoE genes. RefSeq genes with the 5% greatest replication timing changes were defined as EtoL and LtoE, whereas those with the least changes (lowest 20%) that maintained replication timing ratio of above 0.5 or below −0.5 were defined as EtoE and LtoL, respectively. (F) Percentage of 2-fold up- or down-regulated genes within LtoE, EtoL, LtoL, and EtoE domains defined in Figure 4G. (G) Summary of expression patterns of genes within LtoE, EtoL, LtoL, and EtoE domains. Definitions are as follows: “Up” and “Down,” above 2-fold up- and down-regulation, respectively; “Unchanged,” below 2-fold up-/down-regulation; “Unchanged Only,” domains with both active and silent genes that do not change by 2-fold; and “Silent Only,” domains with only silent genes in both states. (H) LINE-1 transposable elements are actively transcribed in ESCs but become inactive in NPCs as assayed by RNA-FISH. Mean and standard error of mean (SEM) of the number of RNA-FISH signals per nucleus is shown (*n* = 30 from two biological replicates). The *p*-value was obtained from a two-tailed *t*-test for comparison of two unpaired groups.

Genes that are transcribed are generally early replicating, whereas genes that are late replicating are almost always silent; however, exceptions to this rule have been described [16,47,48]. Importantly, no study has comprehensively examined the changes in gene expression as they relate to changes in replication timing. To address this issue, we analyzed the steady-state levels of annotated gene transcripts before and after differentiation to NPCs using Affymetrix GeneChips. Regardless of whether levels, density, or number of active genes were examined, either at the level of domains (Figure 5A and 5B) or individual genes (Figure 5C and 5D), both differentiation states clearly showed a similar positive correlation between early replication and transcription. Consistent with previous findings across a portion of the *Drosophila* genome [25], this positive correlation was greater when integrated over large regions (approximately 600 kb for ESCs and NPCs vs. 180 kb in *Drosophila*; not shown). The maintenance of this statistical relationship during differentiation can be accounted for by the directionality of transcriptional changes within each domain (Figure 5E and 5F). At the level of individual genes, LtoE genes were mostly up-regulated, whereas EtoL genes showed a weak tendency to be down-regulated. At the level of domains, among those domains that contained at least one RefSeq gene (National Center for Biotechnology Information [NCBI] most well annotated; http://www.ncbi.nlm.nih.gov/RefSeq/), the majority of LtoE domains contained only up-regulated genes, whereas EtoL domains contain mostly down-regulated or unchanged genes (Figure 5G). Note that because most genes remain early replicating, the overall trend in gene expression changes is dominated by this EtoE group, with a similar amount of genes activated or repressed during this period.

However, there were many exceptional genes, including classes that were up-regulated within EtoL or LtoL domains. In fact, we even detected a weak association of gene activation within LtoL domains (Figure 5E, 5F, and 5G) that leads to a higher probability of very late genes being expressed after differentiation (Figure 5C vs. 5D; *p* = 8.0E−06 when genes with replication timing ratio of less than −1 were compared using a two-tailed Fisher exact test). Moreover, our results demonstrate that there is little or no relationship between replication timing and the probability of transcription for genes replicated throughout nearly the entire first half of S-phase (Figure 5C and 5D); genes with greater than 0.5 replication-timing ratios have an equal probability of transcription, whereas those with negative replication-timing values show a clear positive correlation between their replication time and their probability of being expressed. It is important to keep in mind that these analyses are limited by the fact that noncoding and transposon transcription are not taken into account and are difficult to accurately assess [49]. In fact, we found that LINE-1 tranposons are expressed in mESCs, as recently shown for hESCs [50], and that these active LINE-1 elements are then repressed during the course of differentiation (Figure 5H). These results are consistent with a recent report that revealed global transcriptional activity in ESCs that was down-regulated during differentiation, which was most prominent in intergenic and intronic regions including repetitive sequences such as LINE-1 [49]. Because EtoL domains are exceptionally enriched for LINE-1 elements (Figure 4G), it is possible that LINE-1 silencing takes place within the EtoL domains, something that is currently impossible to verify since the elements are so highly repetitive and widespread. In short, there is a general trend for replication timing and transcription to change coordinately, but given the number of exceptional examples, it is highly unlikely that there is a direct relationship between the two.

#### Replication timing correlates with active, but not repressive, histone marks.

**Figure 6:**
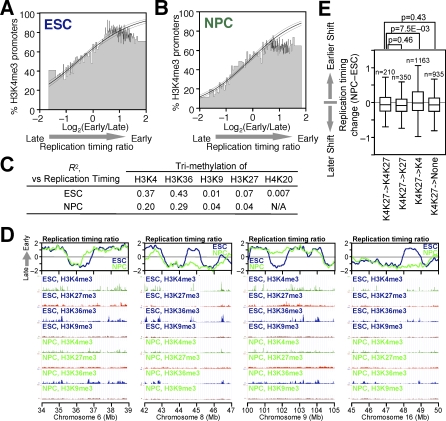
Relationship between Replication Timing and Histone Modifications (A and B) Correlation between replication timing and H3K4me3 of RefSeq gene promoters. The graphical format is the same as in Figure 5C and 5D, except that the heights of bins represent the percentages of H3K4me3-positive genes within each bin. Logistic regression (inner line) and 95% confidence intervals (outer lines) reveal a clear correlation in both ESCs (A) and NPCs (B) (*p* < 2 × 10^−16^ by the Likelihood Ratio test). H3K4me3 data were based on Mikkelsen et al [51]. (C) Relationship between replication timing and histone modifications at the level of replication domains. Densities of different histone modifications (total intensity/domain size) based on a ChIP-Seq study [51] were calculated for all replication domains in a given state (ESC or NPC) and Pearson *R* ^2^ values between replication timing and different histone modification densities were obtained. (D) Comparison of replication timing and different histone modifications in four exemplary 5-Mb genomic regions in ESCs and NPCs. (E) Box plots showing the distribution of replication-timing changes of “bivalently” modified genes (i.e., K4K27) in ESCs that change to four different modification state (K4K27, K27, K4, or none) in NPCs. Genes that remained “bivalent” showed a distribution similar to the three other classes. The *p*-values were obtained from a two-tailed *t*-test for comparison of two unpaired groups.

We also examined the relationship between replication timing and other epigenetic marks that have been analyzed in mESCs and NPCs [51]. A clear correlation between early replication and both lysine 4 tri-methylation of histone H3 (H3K4me3) and H3K36me3 was observed, both at the level of individual genes (Figure 6A and 6B) and when the density of these marks was integrated within the boundaries of each replication domain (Figure 6C). Similar to transcription, a positive correlation was maintained during differentiation. This was expected due to the association of these chromatin marks with transcription [52]. However, there was a significant decrease in the positive correlation to these marks during differentiation (Figure 6C), as well as the overall number of H3K4me3 promoters (Figure 6A and 6B), which is consistent with the recent finding that there is more overall coding and noncoding transcription in ESCs versus NPCs [49]. In contrast, there was little or no relationship between late replication and the repressive marks H3K27me3, H3K9me3, or H4K20me3 in ESCs or NPCs (Figure 6C), which was also evident from visual inspection of representative genomic regions that exhibit changes in replication timing during differentiation (Figure 6D). In fact, a large fraction of genes that changed replication timing during differentiation did not contain H3K27me3 at their promoters in either ESCs or NPCs, which was also true for genes that remained late replicating in both differentiation states (Table S1). Moreover, the lack of correlation to replication timing was confirmed at the level of genes using independently derived H3K27me3 and H3K9me3 microarray data for more than 10,000 promoters in mouse ESCs and NPCs (H3K27me3: *R* ^2^ = 0.004 [53] or 0.01 [54] in ESCs, 0.003 [54] in NPCs; H3K9me3: *R* ^2^ = 0.007 [53] in ESCs; see Table S1). We conclude that replication timing correlates with annotated chromatin marks that reflect transcription, but not repression.

This finding contrasts with a recent report that found a correlation between late replication and H3K27me3 in HeLa cells for the 1% of the genome covered by ENCODE [1]. However, our conclusions are supported by several other observations. First, we find that 87% of promoters marked by H3K27me3 in ESCs are early replicating. Second, disruption of the *Eed* gene, a subunit of the Polycomb complex PRC2, eliminates H3K27me3 in ESCs but did not affect replication timing of several tested genes [55]. Third, LINE-1 elements, which are highly enriched in late replicating DNA, are not enriched for either H3K27me3 or H3K9me3 in ESCs [56]. Differences in our findings could be due to the small fraction of the genome queried by ENCODE regions, or biological differences between ESCs versus HeLa cells, which exhibit a great deal of genetic and epigenetic instability.

#### Replication timing changes are unrelated to the resolution of “bivalency.”

Approximately 2,500 silent, developmentally regulated promoters in ESCs are characterized by a “bivalent” state co-occupied by active (H3K4me3) and repressive (H3K27me3) histone modifications [51,57,58]. Many (but not all) of these promoters resolve to harbor only one of the two modifications upon differentiation, with activated genes harboring H3K4me3, while those remaining silent harbor H3K27me3. To determine whether replication-timing changes reflect the resolution of bivalency, we surveyed a list of 2,658 “bivalent” genes in ESCs (corresponds to 98% of the 2,706 “bivalent” genes in Supplementary Table 3 by Mikkelsen et al [51], for which we could match replication timing values; see Table S1 for their identity). The majority of bivalent genes replicated in the first half of S-phase in both states (not shown), and there was no obvious relationship between changes in these modifications and replication-timing changes (Figure 6E), demonstrating that resolution of bivalency is not related to replication-timing changes observed upon differentiation.

#### High and low CpG density promoters show distinct behaviors upon entering a late-replicating environment.

**Figure 7:**
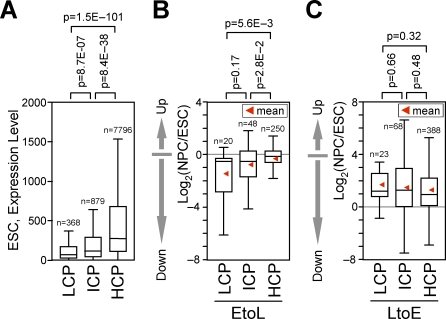
Transcription from Low, But Not High, CpG Density Promoters Is Coordinated with Late Replication (A) Box plots showing the expression level of transcriptionally active (“present”) genes with different promoter CpG density (HCP, ICP, and LCP as defined in [51]), based on Affymetrix GeneChip analysis of RefSeq genes. (B and C) Box plots showing the fold changes in transcription [i.e., log_2_(NPC/ESC)] of LCP, ICP, and HCP genes among EtoL (B) and LtoE genes (C). The *p*-values were obtained from a two-tailed *t*-test for comparison of two unpaired groups.

Given the presence of genes whose expression does not change in parallel with changes in replication timing, an important contribution would be to identify specific classes of promoters distinguished by how they behave transcriptionally upon changes in replication time. Mammalian promoters can be classified based on their CpG density as high, intermediate, and low CpG–containing promoters (HCP, ICP, and LCP, respectively), which are subject to different modes of regulation [51,59]. In fact, among active genes, those with HCP, ICP, and LCPs had the highest, intermediate, and lowest transcript levels, respectively, indicating that HCPs are more strongly expressed than ICP or LCPs (Figure 7A). Interestingly, we found that LCP and ICP genes were generally repressed when residing within EtoL domains, whereas HCP genes were not (Figure 7B). On the other hand, gene activation occurred regardless of promoter CpG density for genes within LtoE domains (Figure 7C), consistent with the hypothesis that a switch to early replication creates a permissive environment for transcription. Moreover, activation of genes within LtoL domains was significantly biased toward HCP genes (not shown). These results suggest that the transcription of CpG-rich, strongly expressed promoters is not coordinated with replication timing upon entering a late-replicating environment.

### Temporal Reorganization Reflects Spatial Reorganization

**Figure 8:**
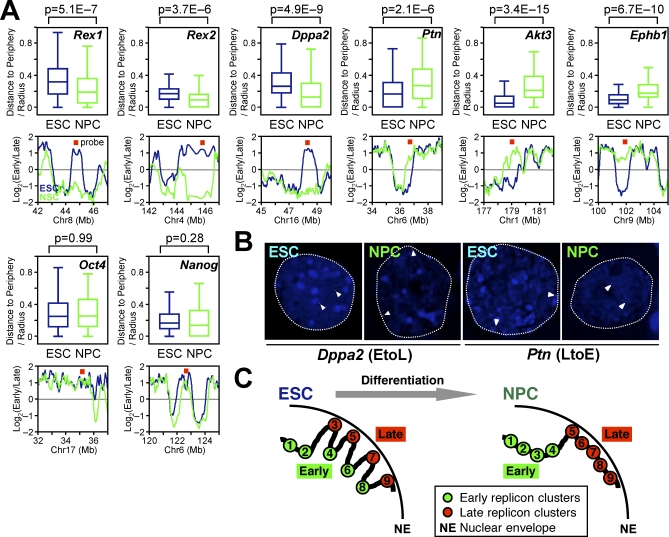
Global Temporal Reorganization of Replication Domains Reflects Spatial Reorganization in the Nucleus (A and B) Analysis of subnuclear positions of eight genomic regions by 3D-FISH in ESCs and NPCs. Box plots show radial distance to the nuclear periphery, where 0 and 1 represents the periphery and the center of the nucleus, respectively. Replication-timing profiles and the probe positions (red squares) are also displayed. Three EtoL domains (*Rex1*, *Rex2*, and *Dppa2* domains) and three LtoE domains (*Ptn*, *Akt3*, and *Ephb1* domains) move toward and away from the nuclear periphery, respectively, upon neural differentiation. Two EtoE domains (*Oct4* and *Nanog*) do not change subnuclear positioning. Comparable results were obtained from two to four biological replicates, and the sum of all experiments is shown. A total of 90–234 alleles were measured per state. (B) Representative FISH photographs (*Dppa2* and *Ptn*). Dotted lines represent the rim of nuclear DAPI signals. Arrowheads represent DNA-FISH signals. (C) A model for higher-order chromosomal organization in the nucleus during neural differentiation (see Discussion). Consider an example of two adjacent isochores, one GC rich (replicon clusters 1–4) and one AT rich (replicon clusters 5–9). In ESCs, replicon clusters with an unusual combination of GC content and gene density (clusters 3, 6, and 8) replicate differently and may be spatially separated from the rest of the isochore. During differentiation, there is an increased influence of isochore sequence features on replication timing, resulting in the temporal consolidation of these deviant domains and alignment of their replication timing to isochores, possibly accompanied by spatial reorganization.

Early replication takes place in the interior of the nucleus, whereas the nuclear periphery is a late-replicating compartment [4,7]. We have shown that this spatiotemporal organization for replication is similar in ESCs and differentiated cells [60,61]. Hence, we investigated the radial subnuclear position (distance to the nuclear periphery) of eight individual genes before and after differentiation, using 3-dimensional (3-D) fluorescence in situ hybridization (FISH) to preserve nuclear morphology. Results (Figure 8A and 8B) revealed that genes within EtoL and LtoE domains moved toward or away from the nuclear periphery, respectively, during differentiation. Subnuclear position changes occurred regardless of whether the replication-timing changes were involved in domain “consolidation” (*Rex1*, *Rex2*, *Dppa2*, and *Ephb1*), “boundary shift” (*Ptn*), or “isolation” (*Akt3*). In contrast, two control EtoE down-regulated genes (*Oct4* and *Nanog*) remained in the nuclear interior during differentiation. These results strongly suggest that the global temporal reorganization of replication domains reflects global 3-D spatial reorganization of chromosomes in the nucleus (Figure 8C). This is significant, given that the current view of spatial genome organization relies primarily on labor-intensive single-gene FISH analysis. We predict that the generation of replication maps for various tissues will create a database of chromosome segments that undergo large changes in 3-D organization during differentiation.

## Discussion

We present the first comprehensive replication-timing study during the course of cellular differentiation. We demonstrate that domains of coordinate replication (“replication domains”) delineate distinct multimegabase units of chromosomes separated by what appear to be large originless transition regions. Replication domain boundaries are remarkably conserved between genetically polymorphic ESCs but change substantially upon neural differentiation, demonstrating that replication profiles are extremely stable epigenetic fingerprints of ESCs and NPCs and likely of other cell lineages. During differentiation, replication domains globally consolidate into fewer, larger, coordinately replicated units. Changes primarily affect domains with an unusual combination of gene density and GC content, which results in an alignment of replication timing to isochore GC content, but not gene density. Temporal reorganization and consolidation of replication domains was accompanied by their spatial reorganization in the nucleus. Replication domain consolidation then, provides a novel and unanticipated concept in chromosome reorganization during differentiation and suggests that studies of replication domain structure and dynamics will provide an important molecular handle on previously impenetrable levels of chromosome organization. Futhermore, smaller replication domains that disrupt the alignment of replication timing to isochores may represent a novel characteristic of the pluripotent state.

### Replication Domains, Nuclear Organization, and Pluripotency

We show that ESCs have substantially more and smaller replication domains and do not show the strong relationship between replication timing and isochore DNA sequence features that has been observed with differentiated cells (Figure 4 and [9–11]). Hence, pluripotent cells have a unique replication domain organization that permits adjacent segments of chromosomes to replicate more discordantly (Figure 8C). What could be the driving force for domain consolidation? One possibility is that the influence of DNA sequences on nuclear organization and replication timing requires sufficient time during G1-phase for reestablishment of subnuclear domains prior to initiation of replication. We have previously shown that replication timing is reestablished at a discrete time point during early G1-phase (termed the timing decision point, TDP) coincident with the repositioning and anchorage of domains in the nucleus [4,8,62,63]. ESCs have a very short G1-phase compared to differentiated cells, including NPCs. Hence, the unique replication-timing program in ESCs could reflect the initiation of replication prior to the completed reestablishment of subnuclear architecture. If true, however, a short G1-phase could not simply lead to a lack of organization but would have to generate an ESC-specific architecture to account for the stability of the replication profile in ESCs. Our results also suggest the possibility that isochore sequence composition and the density of transcription-associated chromatin modifications constitute opposing influences on the replication-timing program. In fact, a larger fraction of the genome (coding and noncoding) is transcribed in ESCs versus NPCs [49], including LINE-1 elements that are enriched in AT-rich isochores (Figure 5H), and we find a higher correlation between replication timing and the density of transcription-associated chromatin marks in ESCs versus NSCs (Figure 6C). Hence, transcription may have a more dominant influence over replication timing in ESCs, whereas isochore sequences dominate in differentiated cells.

### Relationship to Cytogenetic Units of Chromosome Replication

Cytogenetic studies have demonstrated that DNA is synthesized in discrete foci that complete replication within 45–60 min [3–6]. These foci consist of collinear segments of chromosomes that replicate coordinately and remain visibly distinct for many cell generations, suggesting that they represent stable structural units of chromosomes. Since DNA fiber studies show coordinate firing of multiple origins (“replicon clusters”) across hundreds of kilobases [64], replication “foci” likely correspond to replicon clusters. What is the relationship between the molecularly defined “replication domains” described here and these cytogenetically defined units? It is estimated that replication foci/replicon clusters encompass less than 1 Mb and are relatively uniform in size [64,65]. We find that the sizes of replication domains are larger and considerably more heterogeneous (Figure S3). On the other hand, the sizes of domains that change replication timing are smaller and more uniform in size (400–800 kb), indicating that replication domains are made up of variable numbers of smaller units (Figure 8C). Hence, “switching domains” may represent replication foci/replicon clusters, whereas replication domains are groups of replication foci. This model is also consistent with the finding that groups of adjacent replication foci frequently replicate sequentially within 1–2 h of each other [66], which would not be resolved by our methods. Further evidence supporting this model comes from the fine structure of our replication profiles (Figure 1D), revealing reproducible small peaks and valleys that are not distinguished by our segmentation algorithm but are consistent with smaller domains alternating in replication timing by 1–2 h. Examining replication timing at higher resolution, using shorter pulse-labeling periods and finer cell-sorting windows, can test this model. In addition, DNA fiber analyses to elucidate the structure of origins and forks across replication domains will reveal whether subdomains with definable boundaries indeed exist.

We have also discovered that transitions between early- and late-replication domains show kinetics consistent with large originless segments, presumably replicated by a single, unidirectional replication fork. Thus, a significant portion of the mammalian genome may be replicated by unidirectional forks that travel long distance (i.e., several hundred kilobases), in a manner similar to the originless transition between early- and late-replicating segments of the immunoglobulin IgH locus [30]. These regions likely represent sequences vulnerable to DNA damage [39–41] and thus create significant challenges to cells during S-phase. It is also possible that there may be additional regions of unidirectional replication between the smaller and sequentially replicating adjacent replicon clusters discussed above. How such unidirectional regions appear when labeled in situ is currently unknown, but it has been proposed that many of the less intensely labeled replication foci could represent single large replicons [64].

### What Is the Function of a Replication-Timing Program?

DNA replication must be regulated to duplicate the genome faithfully and exactly once per cell cycle. This function is conserved in all cell types, and is regulated by alternating, mutually exclusive periods of the cell cycle during which either prereplication complex formation or initiation can take place, but not both [67]. Based upon our current understanding of this regulation, there is no obvious need for a replication-timing program in order to carry out the task of genome duplication. Since the correlation between replication timing and transcription is so compelling, and since chromatin is assembled at the replication fork, with different types of chromatin assembled at different times during S-phase [68], a popular model is that replication timing facilitates the propagation of chromatin states during DNA synthesis [16,47,48]. This model predicts that changes in replication timing should accompany changes in chromatin states during development. However, the degree to which such changes occur has not been rigorously investigated, and some comparative studies have concluded that such differences are relatively rare [9,12,13]. What has been sorely lacking is an analysis of dynamic changes in replication timing occurring during the course of differentiation, which is what we have addressed in this report. Our findings make several important contributions to our understanding of the developmental regulation of replication timing.

First, we show that replication-timing changes affect a sizeable portion of the genome. In fact, 20% of the genome was significantly affected during the conversion of ESCs to NPCs, so it is reasonable to presume that more than 20% is affected during the many cell fate changes throughout development. Our results also identify sequence characteristics of segments that change. These “switching domains” are almost always above a certain AT/LINE-1 density, and have an unusual combination of gene density and GC content. Hence, the finding that human Chromosome 22 shows only 1% differences in replication timing between lymphoid cells and fibroblasts is expected since this is the earliest replicating human chromosome with extremely high GC content [10]. Importantly, our results suggest that a discrete fraction of the genome undergoes changes in replication timing. It will be interesting to determine whether the sequence characteristics defined here also define switching domains during all cell fate transitions or only transitions during early embryogenesis.

Second, we demonstrate that there is no relationship between replication timing and transcription during early S-phase. Because we queried the entire genome, rather than a fraction of the genome or only coding sequences, replication-timing values for each gene are proportional to their time of replication during S-phase. Thus we can estimate that genes replicated during the earliest 40% of S-phase have an equal probability of being expressed (Figure 5C and 5D). Interestingly, early-middle S-phase corresponds closely to the time at which the spatial distribution of DNA replication sites changes dramatically in these cells, from a “euchromatic” to a “heterochromatic” pattern [60,69]. These results suggest that changes in replication timing are not accompanied by transcription changes unless they take place within a certain window of S-phase, or when replication changes are accompanied by changes in subnuclear position. In other words, the period of replication-timing change during S-phase may be more important than the degree of change.

Third, we demonstrate that transcription from CpG-rich promoters (HCP) is not significantly reduced in parallel with a shift into a late-replicating environment, unlike CpG-poor promoters. Since HCPs are considerably stronger than CpG-poor promoters (ICP and LCP) (Figure 7A), this suggests that strong promoters in general may be regulated independently of changes in replication timing. The ability of strong promoters to overcome chromatin repression has been observed in other contexts [70–75] and may provide an explanation for at least some of the exceptional, late-replicating, and expressed genes. Moreover, expression of some genes in *Drosophila* actually requires a heterochromatin environment, although we currently have no means to identify such genes in our dataset [76,77]. In any case, our results suggest that genes with different promoter structures behave differently upon changes in replication timing.

### Replication Profiles as Fingerprints of Cell Identity

Regardless of the role of replication timing, our results demonstrate that replication profiles are stable and representative of cell state. Moreover, differences between these profiles occur at the level of several hundred kilobase units and correspond to changes in subnuclear position. Our discovery that replication-timing domains consolidate and align to isochore sequence properties during differentiation reveals a novel and unanticipated property of chromosome behavior. Hence, it is possible that establishing replication maps will generate a database of chromosome segments that undergo large changes in organization during differentiation as well as changes in the locations and polarities of replication forks that must travel long distances between replication domains.

## Materials and Methods

### ESC/iPS cell culture, neural differentiation, and BrdU labeling.

D3 [34], 46C [36], and TT2 [35] are male ESC lines with a normal karyotype that were cultured in the presence of LIF (leukemia inhibitory factor) as described [42]. iPS cells [46] were cultured in the same way as ESCs in gelatinized flasks. D3 ESCs were differentiated as embryoid bodies in a conditioned medium as described [42], and NPC samples were collected after 9 d of differentiation. The 46C and TT2 ESCs were differentiated in adherent monolayer culture as described [36], and NPC samples were collected after 6 d (46C) or 9 d (TT2) of differentiation. For BrdU labeling, cells were incubated in the presence of 50 μM BrdU for 1 or 2 h, washed twice with ice-cold PBS, trypsinized, and fixed in 75% ethanol as described [22].

### Cell cycle fractionation and isolation of BrdU-labeled DNA.

BrdU-labeled, fixed cells were resuspended in PBS containing 1% FBS (2–3 × 10^6^ cells/ml), stained with propidium iodide (50 μg/ml) for 30 min in the presence of RNaseA (0.5 mg/ml), and then sorted into two cell cycle fractions (early and late S) by flow cytometry, as described [22]. Isolation of BrdU-labeled DNA has been described [22].

### Replication timing analysis by microarrays.

Quality control PCR experiments were performed to confirm enrichment of α*-globin*, β*-globin*, and mitochondrial DNA sequences in the expected fractions of immunoprecipitated DNA samples, early S, late S, and both, respectively. To obtain sufficient target DNA for microarray hybridization, immunoprecipitated DNA samples were amplified by whole-genome amplification (WGA) (Sigma, GenomePlex) as described [78]. We confirmed the maintenance of relative enrichment of several known early- and late-replicating genes before and after WGA. Sample labeling, hybridization, and data extraction were performed according to standard procedures by NimbleGen Systems using a mouse whole-genome microarray with one probe every 5.8 kb (Nimblegen Systems, 2006-07-26_MM8_WG_CGH). For all except 46C NPCs, two independent biological replicates were analyzed, for which early- and late-replicating DNA were labeled reciprocally with Cy3 and Cy5 (i.e., dye switch). For comparison of different probe density, a microarray covering portions of mouse Chromosome 6 and 7 with one probe every 100 bp (Nimblegen Systems, 2006-07-17_MM8Tiling_Set15) was hybridized with D3 ESC samples in duplicate.

### Microarray data normalization and replication-timing ratio calculation.

Normalization procedures were done using R/Bioconductor (http://www.r-project.org), whereas various data analyses were done using either R/Bioconductor, Excel (Microsoft), or Spotfire DecisionSite (Spotfire). For each experiment, raw datasets were loess normalized to remove signal intensity–dependent bias and scaled to have the same median–absolute deviation using limma package (R/Bioconductor). From two replicates, we calculated the mean replication-timing ratios for each probe. Mean ratios were used to generate a smoothed profile using local polynomial smoothing (loess) for each chromosome [span = 300,000/(chromosome size)]. Replication timing ratios of 18,679 RefSeq genes were obtained as follows. Briefly, redundancy was removed from a list of 20,509 RefSeq genes (mm8 assembly refflat.txt file from UCSC Genome Browser; http://genome.ucsc.edu) to generate a list of 18,702 nonredundant RefSeq genes on non-chrN_random chromosomes. Loess-smoothed replication-timing ratios of these genes at their transcription start sites were obtained using an R/Bioconductor script. We excluded 23 genes that resided within large gaps in probes (>0.65 kb) to generate the final list of 18,679 RefSeq genes with replication-timing ratios matched. Complete replication-timing datasets for all (384,849) probes are downloadable from our Web site (http://www.replicationdomain.org ) and are graphically displayed on the Web site.

### Transcription analysis by microarrays.

Total cellular RNA was isolated from D3 ESCs or NPCs (three biological replicates per cell state), and steady-state transcript levels were determined by Affymetrix GeneChip microarrays (Mouse Genome 430 2.0), which were highly reproducible (*R* ^2^ > 0.98 between all replicates). After quality control tests [79], datasets were subject to normalization by the Probe Logarithmic Intensity Error algorithm (PLIER) developed by Affymetrix for calculating probe signals. For each Affymetrix “probe set,” signal intensity of the three biological replicates were averaged (i.e., average intensity). Genes are often represented by multiple probe sets. In such cases, the one with the highest total intensity (i.e., sum of ESC and NPC average intensity) was defined as the representative probe set, and the other probe sets were not used. We did so because such highest-intensity probe sets were empirically most consistent with reverse transcriptase (RT)-PCR analysis and can be defined in an objective way. Present (transcriptionally active) and absent (inactive) calls are generated by MAS5.0 (Affymetrix) per replicate per probe set, which results in multiple present–absent calls for a given gene [=3 × (total number of probe sets for a gene)]. We defined “present” genes as those with more than 50% of all their probe set calls being present. A total of 15,143 (81%) of the 18,679 RefSeq genes, for which replication-timing ratios were obtained, were represented on the Affymetrix GeneChip microarrays and were assigned transcription levels and present–absent calls. Validation of transcription array results was evident from previously published transcription analysis under the same condition [80].

### Identification of replication domains and domains that change replication timing.

DNAcopy (R/Bioconductor) is a segmentation algorithm for the analysis of microarray-based DNA copy number data [32]. For identification of replication domains, we applied this method directly to datasets containing mean replication-timing ratios for all probes before loess smoothing. The parameters, *nperm* (number of permutation) and *alpha* (the significance level for the test to accept change-points), were set at 10,000 and 1 × 10^−15^, respectively, which were empirically determined based on how well the resultant segmentation profile traced the loess-smoothed profile. Once determined, these parameters were fixed and used for objective segmentation of all datasets. Although DNAcopy segmentation infrequently fails to identify segments discernible by visual inspection of loess-smoothed profiles, it does so equally for ESC and NPC profiles. Thus, its performance was sufficient to provide objective evidence for replication domain consolidation. Others have reported the superiority of its overall performance to current alternatives [81,82]. The same strategy was used to identify chromosomal domains that change replication timing, except in this case, datasets consisting of replication timing ratio differential (i.e., NPC ratio − ESC ratio) for all probes were used for segmentation. Among the resultant 2,042 segments, we selected 102 EtoL, 102 LtoE, 232 EtoE, and 96 LtoL domains based on the criteria described in Figure 4G.

### Analysis of transitions between replication domains.

We selected the three chromosomes 2, 11, and 16 because we reasoned that they were representative of the genome. Chromosomes 2, 11, and 16 are large-, intermediate-, and small-size chromosomes with gene density that is intermediate, high, and low, respectively. For random selection of replication domain boundaries, we focused on the middle portion of each chromosome, counting all transitions after nucleotide position 40,000,000 on each chromosome until we counted 25 boundaries. As a result, the following regions were analyzed: chr2:40,000,000–75,000,000; chr11:40,000,000–68,000,000; and chr16:40,000,000–65,000,000. Transition regions were defined as regions with large and unidirectional changes in replication timing along the chromosomes on the loess-smoothed curve. The positions at which this unidirectionality stopped were defined as the two “ledges” of a transition region.

### GC and LINE-1 content calculation.

GC and LINE-1 content was calculated based on the UCSC Genome Browser database (gc5base.txt and chrN_rmsk.txt, mm8 assembly; http://genome.ucsc.edu) using the Table Browser function of the UCSC Genome Browser as well as an R/Bioconductor script.

### DNA-FISH.

DNA-FISH was performed essentially as described [62], with some modifications. Briefly, preparation and fixation of cells were done as described [83] to preserve 3-D structure. BAC probes were used for all genes tested, with some genes additionally tested by PCR probes of 8.9–10.2 kb. Digoxigenin (DIG)-labeled probes were generated using the DIG-nick translation mix (Roche, Cat#11745816910). Primary and secondary antibodies used to detect the DIG-labeled probes were sheep anti-DIG-fluorescein (Roche Applied Science, Cat# 11207741910) and rabbit fluorescein anti-sheep IgG (Vector, Cat#FI-6000), respectively. Images were captured with a DeltaVision Image Restoration Microscope System (Applied Precision) attached to an Olympus IX-71 fluorescence microscope equipped with an Olympus PlanApo 100× 1.42 NA oil objective lens. Optical sections were taken with 0.2-μm spacing and were subsequently enhanced using constrained iterative deconvolution process by softWoRx software (Applied Precision). We defined the radius of each nucleus as one half of the largest diameter of DAPI staining, which decreased slightly upon differentiation but was comparable in ESC and NPC (average radius: 5.3 μm in ESC, *n* = 1,250 vs. 4.9 μm in NPC, *n* = 1,339). We then measured the distance from FISH signals to the nearest nuclear periphery, and divided it by the radius to obtain relative radial distance to the nuclear periphery.

### RNA-FISH.

LINE-1 RNA-FISH was performed essentially as described [84]. LINE-1 primer sequences were 5′-TAATACGACTCACTATAGGGGGCTCAGAACTGAACAAAGA-3′ (forward; underline, T7 promoter) and 5′-GCTCATAATGTTGTTCCACCT-3′ (reverse), which amplifies a 1,041-bp fragment of LINE-1 corresponding to portions of ORF2 and the 3′-UTR (L1MdA2; accession number M13002; 7,713 bp). Importantly, this sequence is conserved in other subfamilies of LINE-1. We used genomic DNA for PCR, and the amplified DNA fragment was purified and used for in vitro transcription, followed by reverse transcription to generate a DIG-labeled, single-stranded DNA probe.

## Supporting Information

Figure S1 Validation of Microarray-Based Replication-Timing Analysis by PCR (A) Validation of microarray experiments by PCR. Pairs of immunoprecipitated BrdU DNA samples from early and late S fractions were subject to PCR and mean percent early S-phase values (i.e., [intensity of early fraction]/[intensity of early and late fractions combined]) from six to seven pairs of DNA samples were calculated, as previously described [22]. Genes above and below 50% were classified as early (E) and late replicating (L), respectively. From microarray data, replication-timing ratios of genes were obtained from the loess-smoothed curve at the transcription start sites. Replication-timing ratios above and below zero were classified as early (E) and late replicating (L), respectively. The resultant binary datasets for 18 genes showed a 100% match (18/18) in ESCs and a 94% match (17/18) in NPCs. Note that this binary classification of PCR results forces some genes that actually change replication timing to be not classified as such: for instance, *Crisp1* (later shift), *Cdh2*, *Postn*, and *Mash1* (earlier shift). However, even such subtle changes were detected on the microarray, as shown by the changes in replication timing ratios from ESCs to NPCs.
(B and C) Comparison to two previously published replication-timing analyses using 46C ESCs [22] (B) and OS25 ESCs [23] (C). (B) PCR results from Hiratani et al. [22] were classified as early (E) and late (L) based on the same criteria as in Figure S1A. (C) Genes called E, ME, and M by Perry et al. [23] were classified as early (E), whereas genes called ML and L were classified as late (L). Both studies combined, 91% (82/90) of the PCR results matched those from the microarray.
(51 KB PDF)

Figure S2 Autocorrelation Analysis of Replication-Timing Data Autocorrelation analysis of replication timing in ESCs. The autocorrelation function (ACF) indicates the degree of similarity between neighboring data points. The result illustrates that relatively uniform replication timing extends over large regions of approximately 1 Mb. The *x*-axis shows the distance on the chromosome in megabases as calculated from the interprobe distance.
(36 KB PDF)

Figure S3 Replication Domain Size Distribution (A–C) Size distribution of replication domains in ESCs (A) and NPCs (B), as well as domains that change replication timing ([C] EtoL and LtoE domains). Domains with replication-timing ratios above and below zero were defined as early- and late-replicating domains, respectively. Domains were categorized by their sizes into bins of equal intervals (0.2 Mb) starting from 0–0.2 Mb as the first bin. Insets in (A–C) show domains below 0.4 Mb in bins of equal intervals (40 kb) starting from 0–40 kb as the first bin.
(D) Scatter plots of replication timing versus domain size in ESCs and NPCs.
(67 KB PDF)

Table S1 Replication-Timing Ratio, mRNA Expression, Histone Modifications [51,53,54], and Promoter CpG Classification [51] of 18,702 RefSeq Genes Analyzed in This Study (6.25 MB XLS)

## Abbreviations

ESC: embryonic stem cell
FISH: fluorescence in situ hybridization
HCP: high CpG–containing promoter
ICP: intermediate CpG–containing promoter
iPS cell: induced pluripotent stem cell
LCP: low CpG–containing promoter
mESC: mouse embryonic stem cell
NPC: neural precursor cell
