# Supplementary material for: Global Reorganization of Replication Domains During Embryonic Stem Cell Differentiation
Source: PLoS Biol. 2008 Oct 7;6(10):e245. doi: 10.1371/journal.pbio.0060245 (PMC2561079; doi:10.1371/journal.pbio.0060245)
Supplement: Supplementary file 1 [file pbio.0060245.sg001.pdf]

Supplementary Figure S1 (Hiratani et al)

A

Gene name	PCR Mean %early S ESC	PCR Mean %early S NPC	PCR E or L ESC	PCR E or L NPC	Microarray Log2(Early/Late) ESC	Microarray Log2(Early/Late) NPC	Microarray E or L ESC	Microarray E or L NPC
Rex1/Zfp42	74%	23%	E	L	0.88	-0.94	E	L
Rex2	65%	35%	E	L	1.22	-1.67	E	L
Dppa2	71%	24%	E	L	1.16	-1.30	E	L
Dppa4	68%	25%	E	L	1.17	-1.45	E	L
Crisp1	46%	29%	L	L	-0.76	-1.25	L	L
Trap1a	52%	40%	E	L	0.18	-0.72	E	L
Ephb1	47%	67%	L	E	-1.32	0.64	L	E
Akt3	40%	61%	L	E	-0.97	0.30	L	E
Ptn	38%	62%	L	E	-0.91	0.79	L	E
Cdh2	33%	47%	L	L	-1.23	-0.45	L	L
Postn	32%	47%	L	L	-1.36	-0.45	L	L
Mash1/Ascl1	30%	47%	L	L	-1.03	0.05	L	E
Nanog	80%	65%	E	E	0.62	1.09	E	E
Fgf4	80%	69%	E	E	1.00	0.91	E	E
Oct-4/Pou5f1	76%	78%	E	E	0.73	1.14	E	E
Mmp15	84%	80%	E	E	1.16	0.88	E	E
α-globin/Hba-a1	73%	79%	E	E	1.32	1.20	E	E
β-globin/Hbb-b1	21%	20%	L	L	-0.84	-1.10	L	L
						Matched:	18/18 (100%)	17/18 (94%)

B

Gene name	PCR (Hiratani et al, 46CESC) E or L	Microarray (D3ESC) Log2(E/L)	Microarray (D3ESC) E or L
Lefty2	E	0.85	E
Dmrt1	E	0.66	E
Tdgf1/Cripto	E	0.99	E
Tcfap2c	E	1.07	E
2010004A03Rik	E	1.02	E
Utf1	E	0.84	E
Ptgis	E	1.10	E
Epha2	E	0.53	E
Pcolce	E	0.64	E
Ltbr	E	1.36	E
Ddr1	E	1.06	E
Dll1	E	1.52	E
Mmp15	E	1.16	E
Slc25a10	E	0.68	E
Nfatc1	E	0.71	E
Bmp1	E	1.26	E
Fgfr1	E	1.21	E
Gpr56	E	1.39	E
Rex1/Zfp42	E	0.88	E
Rex2	E	1.22	E
Spp1	E	0.73	E
1700034H14Rik	E	1.14	E
Ctgf	E	1.28	E
Ddx4	E	1.60	E
Rbpms	E	1.33	E
Tfpi	E	1.53	E
Terf1	E	1.18	E
Ptn	L	-0.91	L
Tspan12/Tm4sf12	L	-1.34	L
Postn	L	-1.36	L
Lox	L	-0.82	L
Mtap2	E	-1.15	L
Sox6	E	-1.17	L
Ptgs2	L	-1.12	L
Crisp1	E	-0.76	L
Adcy8	L	-1.29	L
Car8	L	-1.08	L
Sprr2a	L	-1.10	L
A2m	L	-1.00	L
Mme	E	-0.61	L
Cdh4	E	0.41	E
Lmo1	E	-0.15	L
Trap1a	E	0.18	E
Gbp3	E	0.10	E
Nr0b1	L	-0.25	L
Egfr	E	-0.36	L
Col1a2	E	0.24	E

41/47 Matched (87%)

C

Gene name	PCR (Perry et al, OS25ESC) E or L	Microarray (D3ESC) Log2(E/L)	Microarray (D3ESC) E or L
Scl/Tal1	E	0.93	E
Lmo2	E	1.18	E
Gata1	E	0.37	E
Gata2	E	1.23	E
Gata3	E	0.95	E
T-bet/Tbx21	E	1.04	E
Ebf1	L	0.47	E
Pax5	E	1.10	E
Ikaros/Ikzf1	E	1.19	E
NF-E2/Nfe2	E	1.00	E
C/EBPalpha (Cebpa)	E	1.29	E
PU.1/Sfpi1	E	1.21	E
Myb/c-myb	E	1.23	E
Myog	E	1.30	E
Myod1/Myod	E	0.93	E
Myf5	E	-1.34	L
Sox1	E	1.38	E
Sox2	E	0.94	E
Sox3	L	-0.58	L
Irx3	E	0.50	E
Nkx2-2	E	0.98	E
Nkx2-9	E	0.00	E
Msx1	E	0.25	E
Pax3	E	0.44	E
Pax6	E	0.71	E
Pax7	E	0.85	E
Olig2	E	0.93	E
Neurod/Neurod1	L	-0.71	L
Mash1/Ascl1	L	-1.03	L
Math1/Atoh1	E	1.14	E
Neurog1/Ngn1	E	0.97	E
Neurog2/Ngn2	E	0.68	E
Hes1	E	1.23	E
Hes5	E	0.88	E
Oct-4/Pou5f1	E	0.73	E
Nanog	E	0.62	E
Utf1	E	0.84	E
Rex1/Zfp42	E	0.88	E
Foxd3	E	0.85	E
Dppa5/Esg1	E	0.93	E
Tdgf1/Cripto	E	0.99	E
Fgf4	E	1.00	E
Zfp57	E	0.62	E

41/43 Matched (95%)
