# Supplementary material for: Global Reorganization of Replication Domains During Embryonic Stem Cell Differentiation
Source: PLoS Biol. 2008 Oct 7;6(10):e245. doi: 10.1371/journal.pbio.0060245 (PMC2561079; doi:10.1371/journal.pbio.0060245)

Supplementary Figure S2 (Hiratani et al)

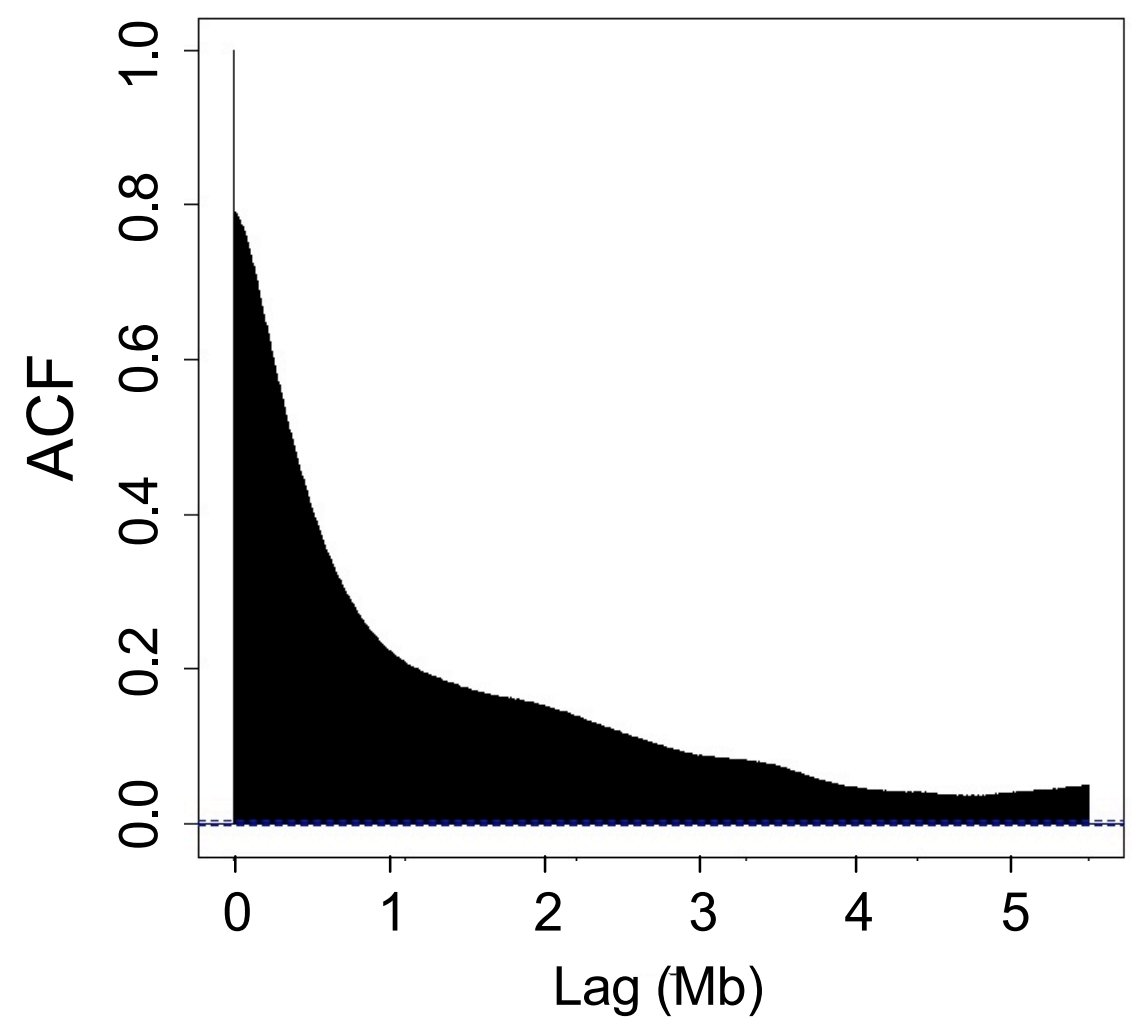

Supplement: Supplementary file 2 [file pbio.0060245.sg002.pdf]
